# Association between tuberculin skin test result and clinical presentation of tuberculosis disease

**DOI:** 10.1186/1471-2334-13-460

**Published:** 2013-10-04

**Authors:** Sara C Auld, Eleanor S Click, Charles M Heilig, Roque Miramontes, Kevin P Cain, Gregory P Bisson, William R Mac Kenzie

**Affiliations:** 1 Division of Tuberculosis Elimination, US Centers for Disease Control and Prevention, Atlanta, USA; 2 Epidemic Intelligence Service, US Centers for Disease Control and Prevention, Atlanta, USA; 3 US Centers for Disease Control and Prevention, Kisumu, Kenya; 4 Department of Medicine, Infectious Diseases Division, Perelman School of Medicine at the University of Pennsylvania, Philadelphia, USA

**Keywords:** Tuberculin test, Miliary tuberculosis, Pulmonary tuberculosis

## Abstract

**Background:**

The tuberculin skin test (TST) is used to test for latent tuberculosis (TB) infection and support the diagnosis of active TB. However, little is known about the relationship between the TST result and the clinical presentation of TB disease.

**Methods:**

We analyzed US TB surveillance data, 1993–2010, and used multinomial logistic regression to calculate the association between TST result (0–4 mm [negative], 5–9 mm, 10–14 mm, and ≥ 15 mm) and clinical presentation of disease (miliary, combined pulmonary and extrapulmonary, extrapulmonary only, non-cavitary pulmonary, and cavitary pulmonary). For persons with pulmonary disease, multivariate logistic regression was used to calculate the odds of having acid-fast bacilli (AFB) positive sputum.

**Results:**

There were 64,238 persons with culture-confirmed TB included in the analysis, which was stratified by HIV status and birthplace (US- vs. foreign-born). Persons with a TST ≥ 15 mm were less likely to have miliary or combined pulmonary and extrapulmonary disease, but more likely to have cavitary pulmonary disease than non-cavitary pulmonary disease. Persons with non-cavitary pulmonary disease with a negative TST were significantly more likely to have AFB positive sputum.

**Conclusions:**

Clinical presentation of TB disease differed according to TST result and persons with a negative TST were more likely to have disseminated disease (i.e., miliary or combined pulmonary and extrapulmonary). Further study of the TST result may improve our understanding of the host-pathogen relationship in TB disease.

## Background

The tuberculin skin test (TST) is primarily used to identify latent tuberculosis (TB) infection in persons who may be at risk of progression to active disease and to support the diagnosis of active TB disease [1,2]. A positive TST result, consisting of measurable skin induration after the injection of tuberculin purified protein derivative, is part of a delayed-type hypersensitivity response of host immune system memory T cells sensitized by prior mycobacterial exposure [3]. However, the TST is an imperfect marker of TB infection and previous reports indicate that 10–25% of persons with active TB disease have a negative TST result [1,4,5].

At the same time, it is well recognized that the host immune system is an important determinant of the clinical presentation of active TB disease, and patients with an immature or suppressed immune system often have faster disease progression and more disseminated disease [6-10]. Likewise, persons with genetic mutations in the interferon-gamma or interleukin-12 cytokine pathways can present with widely disseminated TB disease [11,12]. At the other end of the spectrum, patients with a recovering immune system, such as persons with HIV who are initiating antiretroviral therapy or persons stopping anti-tumor necrosis factor therapy, can have an overexuberant immune response to TB infection characterized by extensive cavitary lung lesions and necrotic lymph nodes [13].

While TST reactivity is recognized to be an indicator of TB infection following exposure to persons with TB disease and has been widely studied in the context of latent TB infection [14], we are not aware of any large studies that describe the pattern of TST results among persons with different clinical presentations of active TB disease. Understanding the association between the TST and clinical manifestations of TB disease may provide insight into the host-pathogen relationship and how factors such as HIV infection may influence that relationship. We analyzed national surveillance data from the United States to explore whether the TST result correlates with differences in the clinical presentation of active TB among a large cohort of persons with bacteriologically-confirmed TB disease.

## Methods

We analyzed reports of persons with culture-confirmed TB in the National Tuberculosis Surveillance System of the Centers for Disease Control and Prevention (CDC) during January 1, 1993 through December 31, 2010. The analysis included persons with a documented TST result, anatomical site of disease, HIV status, and birthplace (US- or foreign-born). Cases of pulmonary TB without a chest radiograph result were excluded to allow for evaluation of the association between radiograph findings and TST result. Reports from California were also excluded because HIV status was not routinely reported from that jurisdiction prior to 2011 [15].

Based on CDC guidelines for the classification of TST reactions, the TST result was divided into categories of 0–4 mm, 5–9 mm, 10–14 mm, and ≥ 15 mm [14]. A TST result of 0–4 mm was considered negative and a result ≥ 5 mm was considered positive. Pearson’s chi-square statistic was used to assess differences in the distribution of TST results for sociodemographic and clinical characteristics. Clinical presentation of disease was defined as one of the following mutually exclusive categories: miliary disease, combined pulmonary and extrapulmonary disease, extrapulmonary only disease, and pulmonary only disease which was further divided into non-cavitary pulmonary disease and cavitary pulmonary disease. A designation of miliary disease was based on either clinical impression or a miliary radiographic pattern on either chest radiograph or CT scan.

Multinomial logistic regression was used to examine the association between TST result category and clinical presentation of disease category and to calculate odds ratios and 95% confidence intervals. Non-cavitary pulmonary disease was the largest clinical presentation category and was used as the referent outcome category. A TST of 0–4 mm (negative) was used as the referent category for TST result. Persons with non-cavitary pulmonary disease with a TST of 0–4 mm served as the comparison group to calculate odds ratios for each of the respective clinical presentation/TST result category combinations (e.g., cavitary pulmonary disease with TST ≥ 15 mm or miliary disease with TST 10–14 mm were all compared to non-cavitary pulmonary disease with a TST of 0–4 mm). We examined the following covariates for effect modification or confounding: sex, age, race and ethnicity (self-designated), HIV status, birthplace, incarceration at the time of diagnosis, homelessness in the 12 months prior to diagnosis, and excessive alcohol or illicit drug use in the 12 months prior to diagnosis. Finally, we conducted an additional analysis restricted to persons with exclusively pulmonary disease who had a documented sputum smear result at baseline. Multivariate logistic regression was used to calculate odds ratios and 95% confidence intervals to quantify the odds of having a positive sputum smear (vs. negative) result for acid-fast bacilli (AFB) for each TST category (TST 0–4 mm referent).

As data were collected as part of routine TB surveillance by the CDC, this analysis was not considered research involving human subjects, and institutional review board approval was not required.

## Results

### Distribution of TST results

**Figure 1:**
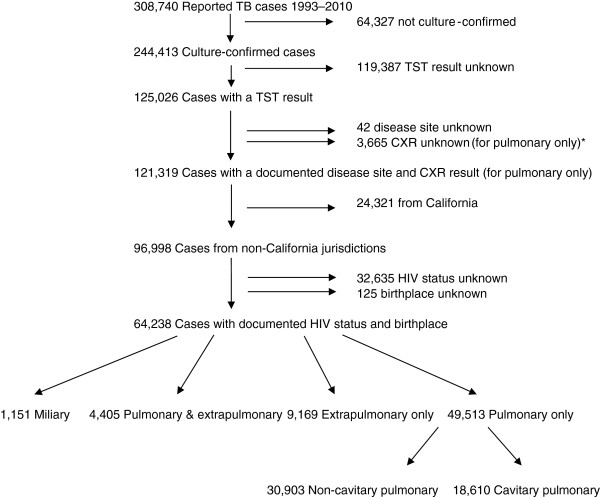
**Selection of United States TB cases reported to CDC during 1993 through 2010 for inclusion in the analysis of the relationship between tuberculin skin test (TST) results and clinical presentation.** *CXR = chest radiograph.

During 1993 through 2010, there were 308,740 cases of tuberculosis reported in the United States, of which 244,413 (79%) were culture-confirmed (Figure 1). Of these cases, 125,026 (51%) had a TST result reported (see Additional file 1, in the online supplement for a comparison of sociodemographic and clinical characteristics of persons with and without a TST result reported, which shows significant differences between persons with and without TST results for all characteristics) of which 64,238 persons with culture-confirmed TB were eligible for inclusion in the analysis. Among these persons, 15.9% had a TST of 0–4 mm (negative), 2.6% had a TST of 5–9 mm, 21.9% had a TST of 10–14 mm, and 59.7% had a TST ≥ 15 mm (Table 1). The proportion of persons with a negative TST was greater in those with age > 45 years, male sex, non-Hispanic white race/ethnicity, who were born in the US, infected with HIV, or who had a positive sputum smear result for AFB at baseline. The distribution of TST results was significantly different among persons with and without HIV: 12.5% of persons without HIV had a negative TST while 38.2% of persons with HIV had a negative TST. Persons with miliary disease and combined pulmonary and extrapulmonary disease also had high rates of TST negativity, 36.7% and 22.9%, respectively, whereas persons with extrapulmonary only, or cavitary pulmonary disease had lower rates of a negative TST with 13.8% and 13.0%, respectively. The distributions of TST results within each sociodemographic and clinical characteristic comparison were statistically significant with a *P*-value < 0.001.

**Table 1:** Tuberculin skin test (TST) result and characteristics of selected culture-confirmed TB cases reported in the United States, 1993–2010 (N = 64,238)

		**TST 0–4 mm**	**TST 5–9 mm**	**TST 10–14 mm**	**TST ≥ 15 mm**	*P*-value*
		**(n,%)**	**(n,%)**	**(n,%)**	**(n,%)**	
**Total**		10,181 (15.9)	1,635 (2.6)	14,049 (21.9)	38,737 (59.7)	
**Age**						
	0–4	52 (9.3)	30 (5.4)	169 (30.2)	308 (55.1)	< 0.001
	5–14	29 (5.7)	9 (1.8)	102 (20.0)	369 (72.5)	
	15–24	658 (7.6)	213 (2.5)	1,891 (21.8)	5,923 (68.2)	
	25–44	4,288 (15.0)	656 (2.3)	6,235 (21.8)	17,450 (61.0)	
	45–64	3,310 (18.0)	496 (2.7)	4,086 (22.2)	10,486 (57.1)	
	65+	1,839 (24.6)	230 (3.1)	1,563 (20.9)	3,831 (51.3)	
**Sex**						
	Male	7,445 (17.5)	1,149 (2.7)	9,483 (22.3)	24,441 (57.5)	< 0.001
	Female	2,731 (12.6)	485 (2.2)	4,563 (21.0)	13,916 (64.1)	
**Race/ethnicity**						
	Hispanic	2,203 (14.7)	404 (2.7)	3,500 (23.4)	8,856 (59.2)	< 0.001
	American Indian	114 (14.0)	20 (2.5)	133 (16.3)	550 (67.3)	
	Asian	673 (6.9)	255 (2.6)	2,153 (22.2)	6,630 (68.3)	
	Black	4,164 (16.7)	529 (2.1)	5,162 (20.7)	15,113 (60.5)	
	Native Hawaiian	46 (6.8)	18 (2.7)	158 (23.3)	455 (67.2)	
	White	2,944 (23.1)	400 (3.1)	2,870 (22.5)	6,546 (51.3)	
**Birthplace**						
	US-born	6,853 (19.8)	881 (2.5)	7,438 (21.4)	19,530 (56.3)	< 0.001
	Foreign-born	3,328 (11.3)	754 (2.6)	6,611 (22.4)	18,843 (63.8)	
**HIV status**						
	Negative	6,997 (12.5)	1,409 (2.5)	12,358 (22.1)	35,131 (62.9)	< 0.001
	Positive	3,184 (38.2)	226 (2.7)	1,691 (20.3)	3,242 (38.9)	
**Clinical presentation of disease**						
	Miliary	422 (36.7)	43 (3.7)	221 (19.2)	465 (40.4)	< 0.001
	Pulmonary/extrapulmonary	1,010 (22.9)	120 (2.7)	869 (19.7)	2,406 (54.6)	
	Extrapulmonary	1,261 (13.8)	207 (2.3)	1,870 (20.4)	5,831 (63.6)	
	Non-cavitary pulmonary	5,061 (16.4)	769 (2.5)	6,805 (22.0)	18,268 (59.1)	
	Cavitary pulmonary	2,427 (13.0)	496 (2.7)	4,284 (23.0)	11,403 (61.3)	
**Sputum smear**						
	Negative	3,350 (13.7)	570 (2.3)	5,138 (21.0)	15,384 (62.9)	< 0.001
	Positive	5,111 (16.4)	854 (2.7)	7,058 (22.6)	18,147 (58.2)	

*Pearson’s chi-square test.

### TST result and clinical category of disease

**Figure 2:**
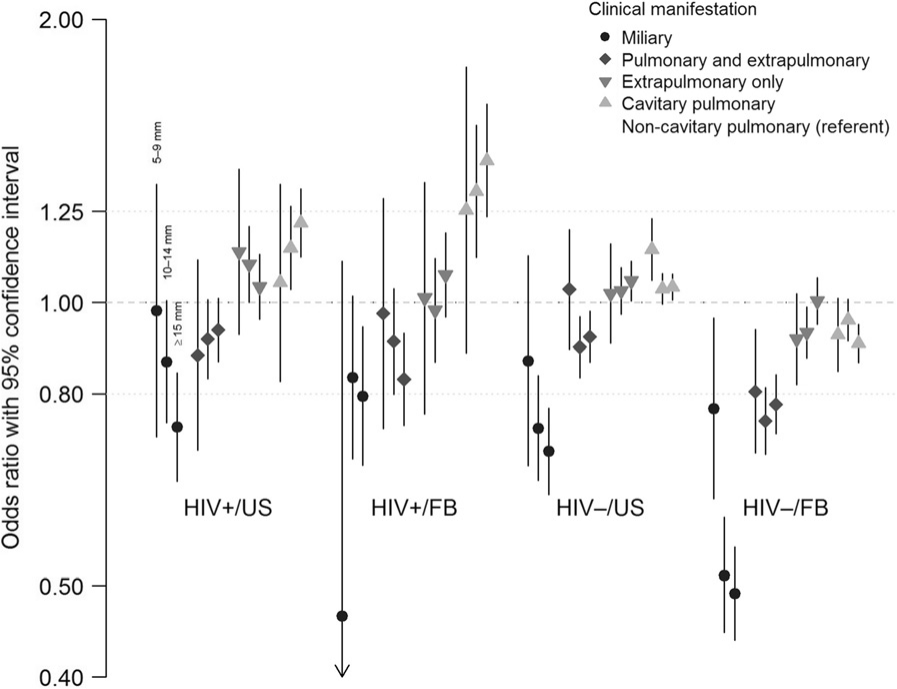
**Association between TST result and clinical presentation of disease relative to a TST of 0–4 mm and relative to non-cavitary pulmonary disease, stratified by HIV status and birthplace and adjusted for age and sex (N = 64,238).** The log of the adjusted odds ratio and their 95% confidence intervals are presented.

In general, we found that persons with a TST ≥ 15 mm were less likely to have miliary or combined pulmonary and extrapulmonary disease, but more likely to have cavitary pulmonary disease relative to non-cavitary pulmonary disease (Table 2 and Figure 2). However, we found statistical interaction between TST result and the covariates of age, sex, HIV status, and birthplace. Because of an *a priori* interest in the potential for HIV status and birthplace to influence immune system priming and immune status, we chose to stratify the analysis by HIV status and birthplace (Table 2 and Figure 2). Age and sex were retained in the subsequent regression models but in the interest of presenting a focused analysis we did not stratify on them. The other covariates did not appreciably impact the regression models.

**Table 2:** Multinomial associations between clinical presentation of disease and tuberculin skin test (TST) result stratified by HIV status and birthplace and adjusted for age and sex among selected culture confirmed TB cases reported in the United States, 1993–2010 (N = 64,238)

		**Miliary disease**		**Pulmonary & Extrapulmonary disease**		**Extrapulmonary only disease**		**Cavitary pulmonary disease**	
		aOR*	95% CI*	**aOR**	**95% CI**	**aOR**	**95% CI**	**aOR**	**95% CI**
**HIV+/US**									
	0–4 mm	Ref		Ref		Ref			
	5–9 mm	0.96	0.47, 1.95	0.72	0.42, 1.22	1.29	0.81, 2.06	1.11	0.64, 1.94
	10–14 mm	0.72	0.51, 1.01	0.81	0.65, 1.01	1.23	1.00, 1.53	**1.36**	**1.07, 1.72**
	≥ 15 mm	**0.50**	**0.37, 0.68**	0.86	0.72, 1.03	1.09	0.91, 1.31	**1.56**	**1.29, 1.90**
**HIV+/FB**									
	0–4 mm	Ref		Ref		Ref		Ref	
	5–9 mm	0.18	0.02, 1.29	0.93	0.49, 1.79	1.01	0.53, 1.94	1.68	0.75, 3.75
	10–14 mm	0.66	0.42, 1.04	0.80	0.60, 1.08	0.96	0.71, 1.28	**1.85**	**1.27, 2.69**
	≥ 15 mm	**0.59**	**0.40, 0.86**	**0.65**	**0.50, 0.84**	1.16	0.92, 1.47	**2.20**	**1.60, 3.02**
**HIV-/US**									
	0–4 mm	Ref		Ref		Ref		Ref	
	5–9 mm	0.77	0.43, 1.38	1.09	0.78, 1.52	1.05	0.79, 1.39	**1.32**	**1.11, 1.56**
	10–14 mm	**0.49**	**0.37, 0.66**	**0.77**	**0.64, 0.91**	1.05	0.92, 1.20	1.09	1.00, 1.18
	≥ 15 mm	**0.41**	**0.32, 0.53**	**0.80**	**0.70, 0.93**	1.11	0.99, 1.24	**1.11**	**1.03, 1.20**
**HIV-/FB**									
	0–4 mm	Ref		Ref		Ref		Ref	
	5–9 mm	**0.56**	**0.34, 0.93**	**0.61**	**0.43, 0.86**	0.82	0.63, 1.06	0.84	0.69, 1.04
	10–14 mm	**0.22**	**0.16, 0.30**	**0.51**	**0.42, 0.62**	**0.85**	**0.74, 0.99**	0.92	0.82, 1.03
	≥ 15 mm	**0.19**	**0.15, 0.25**	**0.56**	**0.48, 0.67**	1.03	0.90, 1.17	**0.81**	**0.73, 0.90**

Non-cavitary pulmonary disease, with the largest number of cases, is the referent clinical category and 0–4 mm (negative) is the referent TST category for all comparisons.

*aOR adjusted odds ratio, *95% CI* 95% Confidence Interval.

Note: Numbers in bold represent significant 95% confidence intervals.

Across all strata, persons with a TST ≥ 15 mm had significantly decreased odds of miliary disease relative to non-cavitary pulmonary disease. (Non-cavitary pulmonary disease will continue to be the referent group for all subsequent comparisons.) The inverse relationship between a positive TST and miliary disease was strongest among persons without HIV for whom those with a TST ≥ 15 mm had 59–81% lower odds of having miliary disease (US-born adjusted odds ratio [aOR] 0.41 [95% confidence interval [CI] 0.32, 0.53]; foreign-born aOR 0.19 [95% CI 0.15, 0.25]) (Table 2 and Figure 2). Persons with HIV who had a TST ≥ 15 mm had 41–50% lower odds of having miliary disease (US-born aOR 0.50 [95% CI 0.37, 0.68]; foreign-born aOR 0.59 [95% CI 0.40, 0.86]). Persons with a TST of 5–9 mm or 10–14 mm also had decreased odds of miliary disease; however, the associations were not consistently statistically significant.

Persons with a positive TST were also less likely to have combined pulmonary and extrapulmonary disease. However, the associations were not as strong as those seen with miliary disease (Table 2 and Figure 2). Persons without HIV who had a TST ≥ 15 mm were significantly less likely to have combined pulmonary and extrapulmonary disease and the strength of association was stronger among foreign-born persons than US-born persons (foreign-born aOR 0.56 [95% CI 0.48, 0.67]; US-born aOR 0.80 [95% CI 0.70, 0.93]). Among persons with HIV, the association between TST result and having combined pulmonary and extrapulmonary disease was significant for foreign-born persons with a TST of ≥ 15 mm (aOR 0.65 [95% CI 0.50, 0.84]) but not for US-born persons (aOR 0.86 [95% CI 0.72, 1.03]).

US-born persons without HIV and all persons with HIV who had a TST ≥ 15 mm were significantly more likely to have cavitary pulmonary disease. The strength of association was greatest for foreign-born persons with HIV where those with a TST of ≥ 15 mm had an aOR of 2.20 (95% CI 1.60, 3.02) for having cavitary pulmonary disease. In contrast, foreign-born persons without HIV who had a positive TST were less likely to have cavitary pulmonary disease (aOR 0.81 [95% CI 0.73–0.90] for TST ≥ 15 mm).

With regard to extrapulmonary only disease, there were no consistent differences between the odds of extrapulmonary disease and non-cavitary pulmonary disease by TST result.

### TST result and sputum smear positivity

Among persons with exclusively pulmonary disease, 50% of those with non-cavitary disease and 83% of those with cavitary disease had a positive sputum smear (Table 3). For persons with non-cavitary pulmonary disease, the odds of having a positive sputum smear result for AFB were significantly decreased among those with a TST ≥ 10 mm. Foreign-born persons with HIV who had a TST ≥ 15 mm had half the odds of having a positive sputum smear when compared to those with a negative TST (aOR 0.50 [95% CI 0.39–0.65]). However, among persons with cavitary pulmonary disease, TST had no consistent association with sputum smear status.

**Table 3:** Association between TST result and sputum smear result for AFB among persons with culture-confirmed pulmonary TB, stratified by HIV status and birthplace and adjusted for age and sex (N = 46,680)

		**Non-cavitary pulmonary disease**				**Cavitary pulmonary disease**			
		**Total**	**Smear-positive (%)**	aOR*	95% CI*	**Total**	**Smear-positive (%)**	**aOR**	**95% CI**
**HIV+/US**									
	0–4 mm	1,073	651 (61)	Ref		183	135 (74)	Ref	
	5–9 mm	79	44 (56)	0.78	0.49, 1.24	16	14 (88)	2.66	0.58, 12.27
	10–14 mm	603	317 (53)	**0.72**	**0.59, 0.88**	139	117 (84)	1.90	1.08, 3.35
	≥ 15 mm	1,182	545 (46)	**0.56**	**0.47, 0.66**	320	245 (77)	1.15	0.75, 1.75
	Sub-total	2,937	1,557 (53)			658	511 (78)		
**HIV+/FB**									
	0–4 mm	522	325 (62)	Ref		62	48 (77)	Ref	
	5–9 mm	36	24 (67)	1.20	0.59, 2.47	7	4 (57)	0.37	0.07, 1.86
	10–14 mm	267	140 (52)	**0.67**	**0.50, 0.91**	61	37 (61)	0.45	0.20, 1.00
	≥ 15 mm	483	217 (45)	**0.50**	**0.39, 0.65**	130	102 (78)	1.03	0.49, 2.16
	Sub-total	1,308	706 (54)			260	191 (73)		
**HIV-/US**									
	0–4 mm	2,051	1,195 (58)	Ref		1,430	1,232 (86)	Ref	
	5–9 mm	285	159 (56)	0.89	0.69, 1.14	273	236 (86)	0.99	0.68, 1.44
	10–14 mm	2,759	1,504 (55)	**0.84**	**0.74, 0.94**	2,137	1,795 (84)	**0.78**	**0.64, 0.95**
	≥ 15 mm	7,576	3,745 (49)	**0.69**	**0.62, 0.76**	5,960	5,027 (84)	**0.80**	**0.68, 0.95**
	Sub-total	12,671	6,603 (52)			9,800	8,290 (85)		
**HIV-/FB**									
	0–4 mm	831	468 (56)	Ref		600	482 (80)	Ref	
	5–9 mm	314	159 (51)	0.78	0.60, 1.02	180	150 (83)	1.23	0.79, 1.91
	10–14 mm	2,702	1,338 (50)	**0.74**	**0.63, 0.87**	1,790	1,466 (82)	1.10	0.87, 1.39
	≥ 15 mm	7,992	3,626 (45)	**0.62**	**0.54, 0.72**	4,637	3,754 (81)	1.03	0.83, 1.28
	Sub-total	11,839	5,591 (47)			7,207	5,852 (81)		
**Total**		28,755	14,457 (50)			19,925	14,844 (83)		

Probability modeled is of sputum AFB smear positivity.

*aOR adjusted odds ratio, *95% CI* 95% Confidence Interval.

Note: Numbers in bold represent significant 95% confidence intervals.

## Discussion

In this analysis of TB cases in the United States, 15.9% of persons with culture-confirmed TB had a negative TST result, and clinical presentation of disease differed by TST result. Overall, persons with a negative TST result were significantly more likely to have miliary or combined pulmonary and extrapulmonary disease, which is consistent with reports from smaller cohorts [1,16,17]. At the same time, persons with a positive TST were typically more likely to have cavitary pulmonary disease as compared to non-cavitary pulmonary disease. A similar relationship has been seen in a rabbit model of aerosolized TB infection whereby more cavities were present in rabbits with strong tuberculin reactions [18]. Importantly, the associations between TST result and clinical presentation of disease were substantially impacted by HIV status and birthplace.

We found that half of persons with non-cavitary pulmonary disease had positive sputum smear results, and this smear positivity was significantly associated with having a negative TST. This association between sputum smear result and TST result was not seen in persons with cavitary pulmonary disease. Our findings support the notion that there may be several mechanisms for the buildup of sufficient bacteria to be visualized by smear microscopy, and smear positive disease in the absence of cavities may be associated with some aspect of immune function that is assayed by the TST [19]. Additionally, several recent studies suggest that *Mycobacterium tuberculosis* (*M. tuberculosis*) benefits from a more active immune response and postulate that direct engagement of *M. tuberculosis* with the human immune system favors cavity formation thereby increasing the likelihood of subsequent aerosol transmission [20-23]. Taken together with our results, this suggests a complex interaction between *M. tuberculosis* and the host immune system that results in different disease manifestations and potential for transmission.

Our finding that HIV status and birthplace impacted the association between TST result and clinical presentation of disease is noteworthy. Associations between TST result and clinical presentation were generally consistent across all strata with the exception of cavitary pulmonary disease. Persons with HIV and US-born persons without HIV who had a TST ≥ 15 mm were significantly more likely to have cavitary pulmonary disease while foreign-born persons without HIV who had a TST ≥ 15 mm were significantly less likely to have cavitary pulmonary disease.

The basis of these differences according to birthplace is not known; however, US- and foreign-born persons with TB in the US differ in several notable respects. One difference in these populations is in the likelihood of previous exposure to *M. tuberculosis* complex. Most foreign-born cases of TB in the US are among persons from countries with high rates of TB and thus potential for repeated exposure in their country of origin [24,25]. Additionally, BCG vaccination as a child is virtually universal among immigrants to the US from medium and high TB burden countries. Thus, foreign-born persons who develop TB in the US are substantially more likely to have had prior exposure to mycobacteria (TB and/or BCG) with resultant sensitization of their immune system and the potential for pre-existing immune function directed against mycobacteria. This immune priming may serve to limit more disseminated forms of TB disease (i.e., miliary and combined pulmonary and extrapulmonary), in a manner potentially analogous to BCG vaccination where vaccinated children have decreased incidence of disseminated disease.

Another potential difference between US- and foreign-born persons may be the timing of disease relative to infection. Disease among US-born persons is more often associated with recent transmission whereas TB among foreign-born persons in the US is thought to be primarily due to reactivation of latent TB [24,26,27]. Hence, cavitation may represent a vigorous, but locally damaging immune response more commonly associated with recent infection. The lower risk of cavitation seen among foreign-born persons without HIV who had a positive TST may represent a “survivor effect” related to different disease manifestations in the setting of reactivation disease. It is also possible that US- and foreign-born persons have differences in their likelihood of undergoing TB screening or different social or nutritional factors that impact both their immune response and their presentation of disease [28,29].

This analysis utilized cross-sectional data and so we could not determine the relative timing of clinical disease presentation and TST result. Prospective studies are needed to determine whether the immune response represented by the TST is a driver of clinical disease presentation or a consequence of infection where, for example, greater presence of mycobacteria may trigger a larger TST response. Although numerous reviews cite disseminated infection as a potential cause of a negative TST in the setting of active disease, it is also possible that disseminated infection occurs as a result of a diminished or impaired host immune response as assayed by the TST [1,3,30].

By limiting our analysis to persons with a documented TST result, we excluded nearly half of the TB cases reported in the United States during the study period. Statistically significant differences were found between the included and excluded populations for all sociodemographic and clinical variables. Therefore, there is a possibility that the population studied was not representative of the entire US surveillance cohort. Nevertheless, we were still able to include a very large cohort of persons with culture-confirmed TB. We also limited our analysis to persons with a known HIV status because we found that the relationship between TST result and clinical presentation varied by HIV status. Similarly, data from California, which accounts for approximately 20% of TB cases in the United States, were excluded because HIV results were not routinely reported to CDC. However, results of a sensitivity analysis including cases reported from California were not appreciably different (data not shown).

## Conclusions

Overall, this analysis provides recent population-level data about the relationship between TST result, a marker of host immune response, and the clinical presentation of active TB disease. Our findings suggest that the significance of the TST result may extend beyond its traditional role as a marker of infection and may be relevant to the pathophysiology and presentation of active disease, even among persons without overt immune dysfunction. The differences in site of disease by TST result may indicate that the TST could be a useful adjunct for identifying patients with different underlying immune system susceptibility to and interaction with *M. tuberculosis*. A better understanding of these differences may provide insight into differential responses to vaccine candidates or TB treatment. Our finding that persons with a positive TST result were less likely to have disseminated disease may parallel the effect of BCG immunization which usually results in transient TST positivity and decreased risk of disseminated disease in children [31]. Future vaccine trials may want to consider including both TST result and clinical presentation of TB disease in their study outcomes since immunization may impact the likelihood of cavity formation or of disseminated disease, both of which would have implications for TB transmission and mortality. Broader incorporation of tuberculin skin testing in TB trials and prospective studies may prove informative as part of the ongoing effort to better understand the relationship between the immune system and *Mycobacterium tuberculosis*.

## Competing interests

The authors declare that they have no competing interests.

## Authors’ contributions

SCA, ESC, CMH, RM, KPC, GPB, and WRM substantially contributed to the study conception and design. SCA performed the statistical analyses and wrote the first draft of the manuscript. CMH provided statistical oversight and guidance. ESC, CMH, RM, KPC, GPB, and WRM critically revised the manuscript for important intellectual content. All authors have read and approved the final version of the manuscript.

## Pre-publication history

The pre-publication history for this paper can be accessed here:

http://www.biomedcentral.com/1471-2334/13/460/prepub

## Supplementary Material

Additional file 1 Table with a comparison of culture-confirmed TB cases reported in the United States from 1993 through 2010 by availability of tuberculin skin test (TST) result and odds (univariate) of having a TST result reported (N = 244,413). Click here for file
