# Supplementary material for: Association between tuberculin skin test result and clinical presentation of tuberculosis disease
Source: BMC Infect Dis. 2013 Oct 4;13:460. doi: 10.1186/1471-2334-13-460 (PMC3851915; doi:10.1186/1471-2334-13-460)
Supplement: Additional file 1 — Table with a comparison of culture-confirmed TB cases reported in the United States from 1993 through 2010 by availability of tuberculin skin test (TST) result and odds (univariate) of having a TST result reported (N = 244,413). [file 1471-2334-13-460-S1.pdf]

ONLINE DATA SUPPLEMENT:

Association between Tuberculin Skin Test Result and Clinical Presentation of Tuberculosis Disease

Table E1. Comparison of culture-confirmed TB cases reported in the United States from 1993 through 2010 by availability of tuberculin skin test (TST) result and odds (univariate) of having a TST result reported (N=244,413).

	No TST result reported (n, %)	TST result reported (n, %)	Odds Ratio (95% Confidence Interval)	*P*-value*
**Total**	119,387 (48.9)	125,026 (51.2)	N/A	N/A
**Age**				
0–4	651 (23.6)	2,111 (76.4)	2.73 (2.50,2.99)	
5–14	445 (23.0)	1,489 (77.0)	2.82 (2.54,3.14)	
15–24	7,413 (32.3)	15,518 (67.7)	1.77 (1.71,1.82)	<.001
25–44	40,694 (45.7)	48,272 (54.3)	Ref	
45–64	35,400 (50.7)	34,454 (49.3)	0.82 (0.80,0.84)	
65+	34,691 (60.0)	23,147 (40.0)	0.56 (0.55,0.58)	
**Sex**				
Male	76,484 (49.0)	79,500 (51.0)	Ref	.015
Female	42,872 (48.5)	45,487 (51.5)	1.02 (1.00,1.04)	
**Race/ethnicity**				
Hispanic	26,492 (45.2)	32,061 (54.8)	1.35 (1.32,1.38)	
American Indian	1,453 (46.8)	1,649 (53.2)	1.27 (1.18,1.36)	
Asian	23,424 (46.8)	26,653 (53.2)	1.27 (1.24,1.30)	<.001
Black	37,617 (50.5)	36,877 (49.5)	1.09 (1.07,1.12)	
Native Hawaiian	549 (31.3)	1,204 (68.7)	2.45 (2.21,2.71)	
White	28,792 (52.8)	25,795 (47.3)	Ref	
**Birthplace**				
US-born	65,912 (51.5)	62,039 (48.5)	Ref	<.001
Foreign-born	52,610 (45.7)	62,630 (54.4)	1.27 (1.25,1.29)	
**HIV status**				<.001
Negative	37,587 (39.6)	57,373 (60.4)	Ref	

Positive	17,272 (63.5)	9,948 (36.6)	0.38 (0.37,0.39)	
Unknown	64,528 (52.8)	57,705 (47.2)	0.59 (0.58,0.60)	
**Clinical category of disease**				
Miliary	2,964 (60.6)	1,925 (39.4)	0.57 (0.54,0.61)	
Pulmonary/extrapulmonary	8,462 (52.9)	7,535 (47.1)	0.78 (0.76,0.81)	<.001
Extrapulmonary	22,710 (55.6)	18,174 (44.5)	0.70 (0.69,0.72)	
Non-cavitary pulmonary	54,625 (46.8)	62,193 (53.2)	Ref	
Cavitary pulmonary	23,958 (43.2)	31,492 (56.8)	1.16 (1.13,1.18)	
**Sputum smear**				
Negative	38,228 (44.2)	48,191 (55.8)	Ref	<.0001
Positive	52,163 (47.9)	56,658 (52.1)	0.86 (0.85,0.88)	

*P-value for Pearson's chi-square test.
